# Salvage After Patient‐Driven Delayed Conversion From External Fixation to Intramedullary Nailing in an Open Tibial Shaft Fracture: A Case Report

**DOI:** 10.1155/cro/1572775

**Published:** 2026-06-09

**Authors:** Md Sulaiman

**Affiliations:** ^1^ Department of Orthopaedics, Trauma General Hospital and Diagnostic Center, Brahmanbaria, Bangladesh

**Keywords:** external fixators, fracture fixation intramedullary, open fractures, tibial fractures, treatment outcome

## Abstract

**Background:**

Open tibial shaft fractures require timely management of both skeletal instability and soft‐tissue injury. Temporary external fixation is useful for early stabilization, but prolonged retention without progression to definitive fixation may increase the risk of delayed union and other complications. This case illustrates a patient‐driven delay from the preferred staged pathway and subsequent salvage after restoration of mechanical stability and biological support.

**Case presentation:**

A 40‐year‐old man sustained a Gustilo–Anderson Type II open fracture of the right tibial shaft with an associated fibular fracture following a road traffic accident. He presented approximately 40 min after injury with a clean 2 cm wound over the anteromedial midleg and intact distal neurovascular status. Immediate preoperative radiographs before initial stabilization were unavailable because the case was reviewed retrospectively. After resuscitation and optimization, irrigation, debridement, primary wound closure, drain placement, and monolateral external fixation were performed on November 22, 2024. Serial follow‐up radiographs showed maintained alignment but limited progression toward union. Early conversion to intramedullary nailing was advised, but the patient initially declined further surgery for personal and socioeconomic reasons. On February 12, 2025, the external fixator was removed and intramedullary interlocking nailing with autogenous bone grafting was performed as a salvage procedure. Follow‐up radiographs showed progressive callus formation and solid union at late follow‐up on March 27, 2026. No postoperative wound complication or deep infection occurred.

**Conclusions:**

This case should not be interpreted as supporting delayed conversion as routine practice. Rather, it illustrates a cautionary salvage scenario in which patient‐driven delay disrupted the preferred staged pathway, and union was achieved only after definitive restoration of stability and selective biological support.

## 1. Introduction

Open tibial shaft fractures remain challenging injuries because they involve both skeletal instability and soft‐tissue compromise, creating a substantial risk of infection and impaired fracture healing [1, 2]. Early treatment therefore aims to achieve timely wound management and stable initial fixation, and temporary external fixation is often used as part of staged management when soft‐tissue care is a priority [3–5]. Once local conditions are suitable, conversion to definitive fixation is generally preferred, with intramedullary nailing widely accepted as an effective option for tibial shaft fractures because it provides stable load‐sharing fixation and supports early mobilization and fracture union [6–10]. In practice, however, the timing of conversion may be influenced by patient‐related factors rather than surgical considerations alone. We report a Gustilo–Anderson Type II open tibial shaft fracture with an associated fibular fracture in which conversion from temporary external fixation to definitive intramedullary nailing was delayed because of patient‐related factors. This case is presented as an illustrative salvage scenario after deviation from the preferred staged pathway, rather than as support for delayed conversion as routine practice.

## 2. Case Report

**Figure 1:**
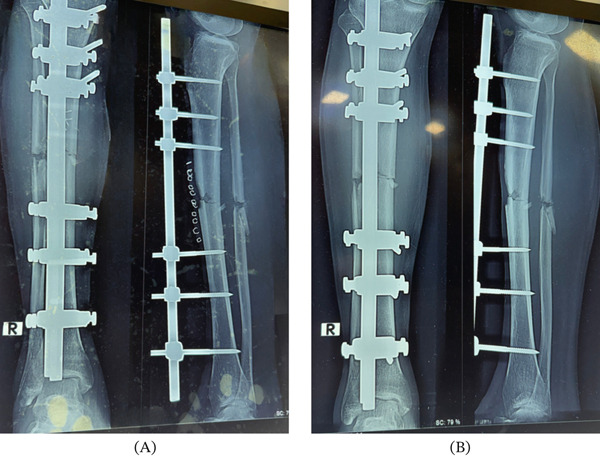
Initial stabilization with external fixation. (A) Anteroposterior and lateral radiographs dated November 29, 2024, showing a right tibial shaft fracture with associated fibular fracture stabilized with a monolateral external fixator after initial wound debridement and primary closure. (B) Anteroposterior and lateral radiographs dated December 27, 2024, showing maintained alignment under external fixation with persistent fracture lines and little callus formation.

**Figure 2:**
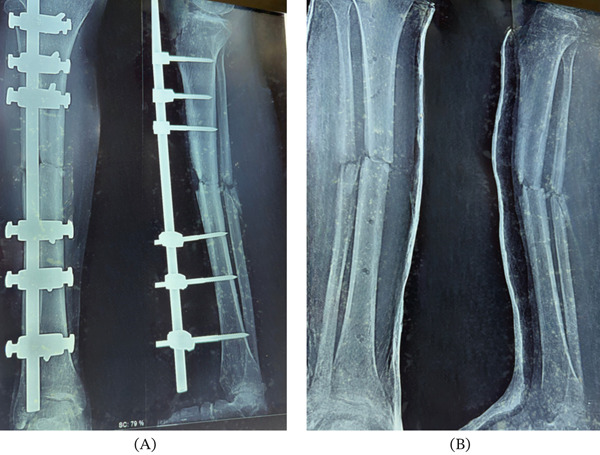
Delayed progression under temporary fixation and preconversion stage. (A) Anteroposterior and lateral radiographs dated January 17, 2025, showing persistent fracture lines and minimal progression toward union while the external fixator remained in situ. (B) Anteroposterior and lateral radiographs dated February 12, 2025, after removal of the external fixator, showing interim cast immobilization before definitive intramedullary nailing.

**Figure 3:**
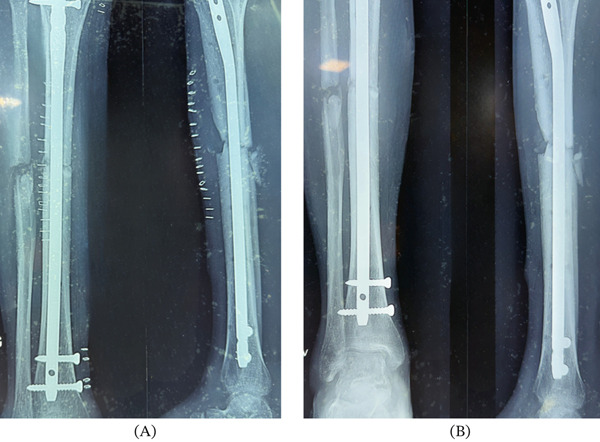
Definitive fixation with intramedullary nailing. (A) Anteroposterior and lateral radiographs dated February 17, 2025, showing intramedullary interlocking nailing with satisfactory alignment and stable fixation. (B) Anteroposterior and lateral radiographs dated March 17, 2025, showing early callus formation and progressive fracture healing.

**Figure 4:**
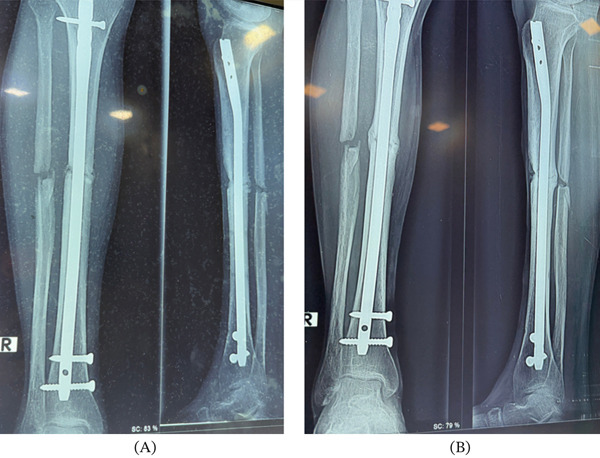
Late healing and final outcome. (A) Anteroposterior and lateral radiographs dated June 13, 2025, showing bridging callus formation and advanced healing. (B) Anteroposterior and lateral radiographs dated March 27, 2026, confirming solid union with cortical continuity and remodeling.

A 40‐year‐old man sustained a Gustilo–Anderson Type II open fracture of the right tibial shaft with an associated fibular fracture following a road traffic accident. He presented to our unit approximately 40 min after injury. Examination revealed a clean 2 cm wound over the anteromedial midleg, and distal neurovascular status was intact. Because this case was identified retrospectively, the immediate preoperative radiographs obtained before initial external fixation could not be retrieved. The fracture pattern was later identified radiographically as transverse fractures of both the tibia and fibula with mild comminution. After initial resuscitation and optimization, the limb was temporarily immobilized in a long leg back slab. On the night of November 22, 2024, approximately 5 h after presentation, the patient underwent irrigation and debridement of the open wound, primary wound closure, drain placement, and monolateral external fixation as the initial definitive treatment. The first follow‐up radiographs, dated November 29, 2024 (Figure 1A), showed the fracture stabilized under external fixation; the skin staples visible on the radiograph were consistent with recent wound closure. Subsequent radiographs dated December 27, 2024 (Figure 1B), demonstrated maintained alignment but persistent fracture lines without significant callus formation. At further follow‐up, radiographs dated January 17, 2025 (Figure 2A), continued to show minimal progression toward union, suggesting inadequate healing under prolonged temporary fixation. At that stage, early conversion to intramedullary nailing was advised once conditions were suitable; however, the patient initially declined further surgery because of personal and socioeconomic considerations. This delay was not clinically intended and represented a patient‐driven deviation from the preferred staged management pathway. He later returned and the external fixator was removed on February 12, 2025. Interim cast immobilization was provided (Figure 2B). On the same day, after renewed counseling regarding the need for definitive stabilization, he consented to surgery and underwent intramedullary interlocking nailing of the tibia with adjunctive autogenous bone grafting. Early postoperative radiographs dated February 17, 2025 (Figure 3A), confirmed satisfactory alignment and stable fixation. Follow‐up radiographs dated March 17, 2025 (Figure 3B), demonstrated early callus formation and progressive healing. Further improvement was evident on radiographs dated June 13, 2025 (Figure 4A), which showed bridging callus and advanced healing. At late follow‐up, radiographs dated March 27, 2026 (Figure 4B), confirmed solid union with cortical continuity and remodeling without implant‐related complications. No postoperative wound complication or deep infection occurred. Clinically, the patient was pain‐free, fully weight‐bearing, and had returned to normal daily activities without functional limitation.

## 3. Discussion

Open tibial shaft fractures remain challenging injuries because skeletal instability and soft‐tissue compromise must be managed simultaneously [1, 2]. Temporary external fixation is a well‐established early stabilization method, particularly when rapid fracture control and wound management are priorities [3–5]. However, in most staged protocols it is intended as a temporary measure rather than a prolonged definitive solution. In the present case, the initial management was appropriate. The patient presented early, underwent irrigation and debridement, primary closure, drain placement, and external fixation, and early radiographs showed acceptable alignment. The clinical concern emerged later, when serial imaging showed limited biological progression toward union despite maintained fracture position.

The main value of this case lies in illustrating a patient‐driven deviation from the preferred pathway and the subsequent salvage required to restore fracture healing. Early conversion from external fixation to intramedullary nailing is generally preferred when soft‐tissue conditions permit, because it provides more durable stability, supports rehabilitation, and creates a more favorable mechanical environment for union [6–10]. In this patient, delayed conversion was not related to infection, implant failure, or persistent soft‐tissue compromise. Instead, it resulted from delayed patient acceptance of further surgery. This distinction is important because the delay should not be interpreted as an intended or recommended treatment strategy.

Contemporary principles of open tibial fracture management emphasize timely progression to definitive fixation when feasible, minimization of unnecessary time in temporary external fixation, and careful respect for both soft‐tissue and fracture biology. Prolonged reliance on temporary external fixation may increase the risk of delayed union, nonunion, pin‐site problems, infection, and fracture‐related infection. In this case, the absence of clinical evidence of deep infection or wound complication was favorable; however, the possibility of infection‐related complications is an important consideration in any open fracture with delayed healing. Fracture‐related infection is now recognized as a distinct and clinically important complication after fracture fixation, and delayed healing in an open fracture should prompt careful clinical assessment for infection before reconstructive decisions are made [11, 12]. Autogenous bone grafting in this case should be interpreted as a salvage measure rather than an innovative or routinely desirable adjunct. By the time definitive surgery was accepted, the fracture had shown limited progression toward union under prolonged temporary fixation. Intramedullary interlocking nailing addressed the mechanical deficit, while autogenous bone grafting was used to support a compromised biological environment [13–15]. This sequence should therefore be understood as correction of a delayed and suboptimal pathway, not as a recommendation for early or unnecessary grafting in open tibial fractures.

The favorable final outcome in this case likely depended on several factors: early presentation, a small and clean wound, timely initial debridement and closure, maintained fracture alignment, absence of postoperative wound complications, and eventual restoration of mechanical stability. These features help explain the successful union, but they do not make delayed conversion a preferred strategy. The case instead emphasizes that when real‐world factors delay ideal care, treatment should be redirected toward accepted principles as soon as the patient accepts definitive management. From an educational standpoint, the value of this case is not the demonstration that union can occur after delayed conversion, which is already consistent with established fracture‐healing principles. Rather, the case highlights three practical points in staged open tibial fracture care. First, patient‐driven delay should be recognized as a deviation from the preferred pathway and should trigger repeated counseling rather than passive observation. Second, prolonged temporary fixation with limited callus progression should prompt reassessment of both mechanical stability and infection‐related risk, including the possibility of fracture‐related infection. Third, when definitive care is delayed, salvage surgery should aim to restore accepted principles of fracture treatment—stable fixation, appropriate soft‐tissue status, and biological support when indicated—without implying that delay or routine grafting is desirable.

This report also highlights the importance of counseling and shared decision‐making. The delay in definitive fixation was influenced by patient‐related and socioeconomic factors. Progress became possible only after renewed counseling regarding the need for stable fixation. In staged trauma care, communication is therefore not separate from treatment; it is part of maintaining an effective and safe treatment pathway. This report has important limitations. It is a single retrospective case and cannot establish causality or support generalizable conclusions. The immediate preoperative radiographs before the initial external fixation could not be retrieved. Objective functional outcome scores were not recorded, and the clinical outcome is therefore described narratively rather than quantitatively. In addition, although no clinical evidence of deep infection was observed, formal infection markers and microbiological data were not available for detailed fracture‐related infection assessment. The findings should therefore be interpreted as illustrative rather than evidence‐generating.

Overall, this case demonstrates that successful union may still be achieved after patient‐driven delayed conversion from external fixation to intramedullary nailing, but only after restoration of appropriate mechanical stability and biological support. The message is not that delayed conversion is acceptable routine practice; rather, the case illustrates salvage after deviation from ideal timing and reinforces the need for timely definitive fixation whenever feasible.

## 4. Conclusion

Early conversion from temporary external fixation to definitive fixation remains the preferred strategy in open tibial shaft fractures when soft‐tissue and systemic conditions permit. This case should not be interpreted as evidence supporting delayed conversion or routine biological augmentation. Its value is illustrative and cautionary: patient‐driven delay can disrupt the preferred staged pathway, and successful salvage depends on returning to accepted principles of fracture care, including reassessment for infection‐related risk, restoration of stable fixation, and selective biological support when indicated. The findings remain limited by the nature of a single retrospective case.

## Funding

No funding was received for this manuscript.

## Consent

Written informed consent was obtained from the patient for publication of this case report and accompanying images.

## Conflicts of Interest

The author declares no conflicts of interest.

## Data Availability

The data that support the findings of this study are available from the corresponding author upon reasonable request.
